# Improvement of Lipotoxicity-Induced Islet *β* Cellular Insulin Secretion Disorder by Osteocalcin

**DOI:** 10.1155/2022/3025538

**Published:** 2022-03-12

**Authors:** Yafang Zhang, Ling Li, Yongze Zhang, Sunjie Yan, Lingning Huang

**Affiliations:** ^1^Department of Endocrinology, The First Affiliated Hospital of Fujian Medical University, No 20 Chazhong Road, Fuzhou, 350004 Fujian province, China; ^2^Diabetes Research Institute of Fujian Province, No 20 Chazhong Road, Fuzhou, 350004 Fujian province, China; ^3^Institute of Metabolic Diseases of Fujian Medical University, No 20 Chazhong Road, Fuzhou, 350004 Fujian province, China

## Abstract

**Background:**

Osteocalcin (OCN) has been proved to be closely related with the development of type 2 diabetes mellitus (T2DM). We aimed to study if OCN could improve the disorder of islet cell caused by lipotoxicity.

**Methods:**

Alizarin red staining was used to investigate the mineralization. Western blotting and ELISA methods were used to measure protein expression. Immunofluorescence staining was used to investigate the protein nuclear transfer.

**Results:**

High glucose and high fat inhibited the differentiation of osteoblast precursors. Overexpression of insulin receptor (InsR^OE^) significantly promoted the Runx2 and OCN expression. The increase of insulin, Gprc6a, and Glut2 by osteoblast culture medium overexpressing insulin receptor was reversed by osteocalcin neutralizing antibody. Undercarboxylated osteocalcin (ucOC) suppressed the lipotoxic islet *β*-cell damage caused by palmitic acid. The FOXO1 from intranuclear to extranuclear was also significantly increased after ucOC treatment compared with the group PA. Knockdown of Gprc6a or suppression of PI3K/AKT signal pathway could reverse the upregulation of GPRC6A/PI3K/AKT/FoxO1/Pdx1 caused by ucOC.

**Conclusion:**

OCN could activate the FOXO1 signaling pathway to regulate GLUT2 expression and improve the insulin secretion disorder caused by lipotoxicity.

## 1. Introduction

Type 2 diabetes mellitus (T2DM) is one of the most common diseases threatening human health [1, 2], and its pathogenesis is complex. The insulin synthesis, processing, secretion disorders, and insulin resistance are the main pathological mechanisms associated with the *β*-cell dysfunction [3]. The pancreatic *β*-cell dysfunction is a focus clinical indication of diabetes by impairing the ability of insulin secretion [4, 5]. Additionally, the lipotoxicity was an indispensable factor modulated by inflammatory mediators and cytokines [6, 7]. It was reported that the lipotoxicity could directly activate the TLR4-JNK pathway cascade in islet *β*-cells and further induce insulin secretion disorder and *β*-cell apoptosis [8]. The oxidative stress, endoplasmic reticulum stress, and inflammation can also lead to dedifferentiation of mature *β*-cells, which is an important factor in functional cell reduction and insulin secretion deficiency in diabetes patients [9, 10]. In addition, chronic exposure to high concentrations of saturated fatty acids, such as palmitic acid (PA), could lead to apoptosis of pancreatic *β*-cells [11–13].

Osteocalcin (OCN) is the bridge between bone metabolism and energy metabolism [13]. After entering the blood circulation, it decarboxylates under the action of vitamin K-related enzymes and exists in the blood circulation in the form of carboxylated incomplete OCN [14, 15]. Some researchers found that there was no abnormal bone metabolism after selective OCN gene knockout in rats [16]. However, fat thickness, insulin level, glucose tolerance, and other metabolic indicators could be changed [17–19]. Meanwhile, OCN may regulate energy metabolism and have endocrine activity [16, 20, 21]. After intervention with exogenous OCN, the fat thickness of mice fed with high fat and high glucose was decreased, but the insulin secretion and sensitivity were increased [22–25]. Previous study showed that OCN treatment improved glucose homeostasis and lipid metabolism [26]. These evidences indicate that OCN may improve T2DM and glucose and lipid metabolism disorders, but the specific mechanism remains unclear.

Some studies have confirmed that OCN can stimulate insulin secretion [27]. In addition, the serum level of OCN in patients with T2DM was reduced [28]. This may further reduce insulin secretion and aggravate the progression of T2DM. However, the specific mechanism of OCN in insulin synthesis and secretion remains unclear. These evidences prove that bone metabolism and glucose metabolism may form endocrine rings and interfere with each other. OCN may become a new target for the treatment of metabolic diseases.

## 2. Materials and Methods

### 2.1. Cell Culture

The MC3T3-E1 cell line was purchased from American Type Culture Collection (ATCC, Manassas, VA, USA). Cells were incubated with a-MEM (Gibco, USA) containing 1% penicillin-streptomycin and 10% heat-inactivated fetal bovine serum (FBS) at 37°C with 5% CO_2_. *β*-TC6 cell line purchased from ATCC was cultured with DMEM containing 10 mmol/L HEPES, 2 mmol/L L-glutamine, 10% FBS, and 25 mmol/L glucoses on the condition of 95% air and 5% CO_2_ at 37°C.

### 2.2. Cell-Induced Differentiation

Cells were plated into a 12-well plate and cultured with a-MEM containing 100 *μ*g/mL ascorbic acid, 10 mM B-glycerophosphate, 1% penicillin-streptomycin, and 10% FBS on the condition of 95% N2 and 5% CO_2_ at 37°C. After different incubation times (1, 3, 7, 14, and 21 days), the cells were used for additional experiments.

### 2.3. Preparation of Solution and Conditioned Medium (CM)

The preparation of osteogenic induction solution was performed as follows. *β*-sodium glycerophosphate solution (10 mM) and ascorbic acid solution (50 mg/L) were prepared with *α*-MEM medium. 1 mL 50 mg/L ascorbic acid solution and 9 mL *β*-sodium glycerophosphate solution (10 mM) were mixed and 100 times concentrated osteogenic induction solution was obtained. After filtering with 0.22 *μ*m filtration, the solution was kept away from light at -20°C.

The preparation of high sugar solution was performed as follows. The glucose solution (25 mmol/L) was prepared with *α*-MEM. After filtering with 0.22 *μ*m filtration, the solution was kept away from light at -20°C.

The preparation of palmitic acid (PA) solution was performed as follows. 0.0307 ɡ PA was added to 3 mL NaOH (0.1 mol/L). After saponification for 30 min at 75°C water bath, the 40 mM PA saponification solution was obtained.

The preparation of undercarboxylated osteocalcin (ucOC) was performed as follows. The ucOC (1 *μ*g/*μ*L) was prepared with DMSO. The ucOC (1 *μ*g/*μ*L) was diluted with PBS when use.

The preparation of CM was performed as follows. The InsR^OE^ MC3TC-E1 cells were induced differentiation for 14 days. The CM was collected and centrifuged at 1500 g for 10 minutes. The supernatant was collected, filtered, and stored at -80°C for use.

### 2.4. Plasmid Construction and Transfection

The InsR ovexpression (InsR^OE^) plasmid was constructed using pDC316-mCMV-EGFP. The primer was designed and synthesized by GenePharma (Shanghai, China). The cells were transfected with vector using transient transfection. The cells were subjected to further experimentation after transfection.

### 2.5. Flow Cytometric Measurement

Cells were seeded in a 6-well plate and cultured on the condition of 37°C and 5% CO_2_. After treatment with PA (20 mM) or ucOC (1 *μ*g/*μ*L) for 4 h, the cells were digested using trypsin. After centrifugation at 8,000 rpm for 10 min, the cells were collected and suspended with cold PBS. Then, cells were incubated with propidium iodide and Annexin V-FITC for 30 min in the dark and analyzed using the Cytomics™ FC500 Flow Cytometer (Beckman, USA).

### 2.6. Immunofluorescence Analysis

*β*-TC6 cells were fixed using paraformaldehyde (4%) for 30 min and then incubated using 0.2% Triton X-100 (20 min). PBS containing 8% BSA was used for blocking. Cells were incubated with anti-FOXO1(1 : 500) or anti-TLR4 (1 : 800) overnight at 4°C. After washing twice with PBS, the cells were incubated with second antibody at room temperature for 1 h. DAPI was used to stain the nuclei for 5 min. A confocal microscope was used to analyze fluorescent images.

### 2.7. qRT-PCR

The total RNA was extracted from the *β*-TC6 cells with the TRIzol reagent (Invitrogen, USA). The reverse transcription was performed according to the previous report [29]. Reverse transcription product (2 *μ*L) was mixed with 10.0 *μ*L of qPCR SYBR® Green Master Mix (A6001, Promega, USA), 0.5 *μ*L of primers, and 7.0 *μ*L nuclease-free water. Subsequently, the ABI 7500 PCR system (ABI, USA) was used to detect the mRNA expression. The 2^-△△Ct^ method was used to calculate mRNA expression [30]. The primers were listed as follows: Runx2: F: 5′-ATGCTTCATTCGCCTCACAAA-3′, R: 5′-GCACTCACTGACTCGGTTGG-3′; OCN: F: 5′-CATGCCAGGTCACCAAAT-3′, R: 5′-GCCCCAAGGCCGCTTCTT-3′; Glut2: F: 5′-GAAGACAAGATCACCGGAACCTTGG-3′, R: 5′-GGTCATCCAGTGGAACACCCAAAA-3′; Insulin F: 5′CCCACCCAGGCTTTTGTCAAACAGC-3′, R: 5′-TCCAGCTGGTAGAGGGAGCAGATG-3′; Actin: F: 5′-AGAGCTACGAGCTGCCTGAC-3′, R: 5′-AGCACTGTGTTGGCGTACAG-3′.

### 2.8. Western Blotting

The protein samples were extracted from the cells with RIPA buffer including the PMSF. The supernatant solution was collected after centrifuging at 8 000 rpm for 10 min. Same amounts of proteins were separated with SDS-PAGE. Then, the proteins were transferred to a PVDF membrane (Sigma, USA). After blocking with TBST, the membrane was incubated with anti-GLUC2, T-AKT, p-AKT, p-FOXO1, T-FOXO1, Pdx1, and *β*-actin (1 : 1500, Abcam, Beijing, China) overnight at 4°C. After washing twice, the membrane was incubated with secondary antibody (antimouse HRP-conjugated antibody, 1 : 2000) for 1 h. Finally, the protein was detected by chemiluminescence with Thermo ECL Substrate (Bio-Rad) and analyzed using the ImageJ software.

### 2.9. Alizarin Red Staining

The cells were fixed using paraformaldehyde (4%) for 20 min. After washing with water twice, the cells were stained using alizarin red staining solution (pH 4.2) for 20 min. After washing with water for 3 times, the cells were captured using a microscope.

### 2.10. TUNEL Staining

*β*-TC6 cells were fixed using paraformaldehyde (4%) for 30 min and washed with PBS. 0.2% Triton X-100 was used to incubate cell for 5 min. The TUNEL staining solution was prepared with TdT enzyme and fluorescent labeling solution (#C1090, Beyotime, Shanghai, China) according to the instruction. The cells were incubated with TUNEL staining solution in the dark at 37°C for 60 min. After washing 3 times with PBS, the cells were observed with a confocal microscope. DAPI was used to stain the nuclei for 3 min.

### 2.11. Statistical Analysis

Data was shown as mean ± SD and analyzed with the SPSS software (20.0, IBM, USA). Student's *t*-test or a one-way ANOVA were performed for data analysis. *P* < 0.05 was believed to be statistically different.

## 3. Results

### 3.1. Induction Time of Osteogenic Precursor Cells

**Figure 1:**
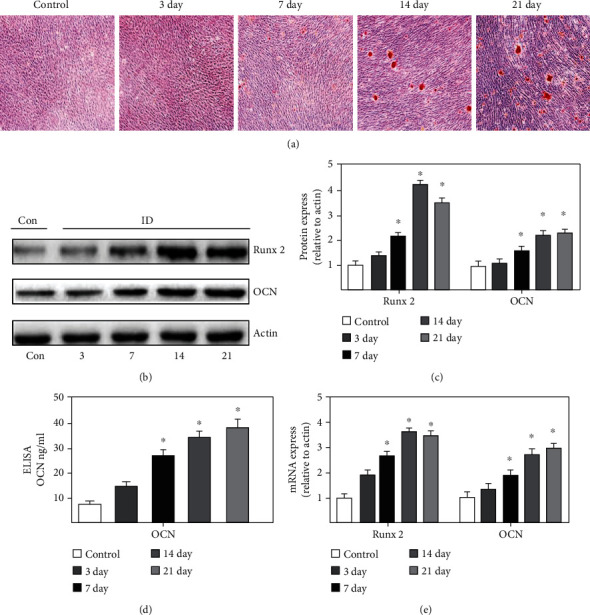
Induction time of osteogenic precursor cells. (a) Mineralization was analyzed through alizarin red staining. (b) The protein expression of Runx2 and OCN was measured through western blotting. (c) The protein expression of Runx2 and OCN was analyzed. (d) The level of OCN was measure with ELISA. (e) The mRNA expression of Runx2 and OCN was detected. Compared with the control group, ^∗^*P* < 0.05. MC3T3-E1 was used in these experiments.

*β*-Sodium glycerophosphate was used as an osteogenic inducer to induce osteoblast differentiation. Alizarin red staining showed that mineralization gradually increased with the extension of differentiation time, and more mineralized nodules were produced at 14 days of differentiation (Figure 1(a)). The protein and mRNA expression of Runx2 and OCN were increased gradually with the extension of differentiation time (Figures 1(b), 1(c), and 1(e)). The increase trend of OCN was also identified with ELISA method, and it was promoted with the increase of differentiation time (Figure 1(d)).

### 3.2. High-Glucose and High-Fat Diet Inhibited the Differentiation of Osteoblast Precursors

**Figure 2:**
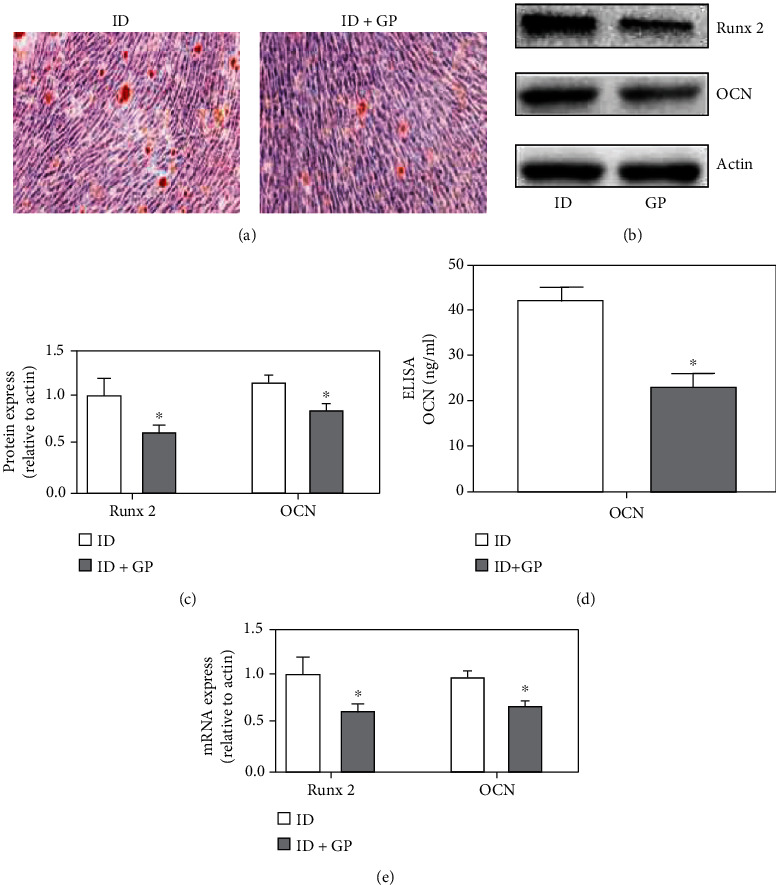
High glucose and high fat inhibited the differentiation of osteoblast precursors. (a) Mineralization was analyzed through alizarin red staining. (b) The protein expression of Runx2 and OCN was measured through western blotting after treatment with ID or ID+GP; (c) The protein expression of Runx2 and OCN was analyzed. (d) The level of OCN was measured with ELISA. (e) The mRNA expression of Runx2 and OCN was detected. Compared with the ID group, ^∗^*P* < 0.05. MC3T3-E1 was used in these experiments.

Based on the data in Figure 1, 14-day incubation was used for induction differentiation (ID). The high-glucose and high-fat model was established with glucose (25 mM) and palmitic acid (100 mM) (GP). We found that mineralized nodules were markedly decreased in the group ID+GP compared with the group ID (Figure 2(a)). In addition, the protein and mRNA levels of Runx2 and OCN were significantly decreased after GP treatment compared with ID administration only (Figures 2(b)–2(e)). Therefore, high-glucose and high-fat diet could inhibit the differentiation of osteoblast precursors.

### 3.3. Effect of Insulin Receptor Overexpression (InsR^OE^) on Runx2 and OCN Expression

**Figure 3:**
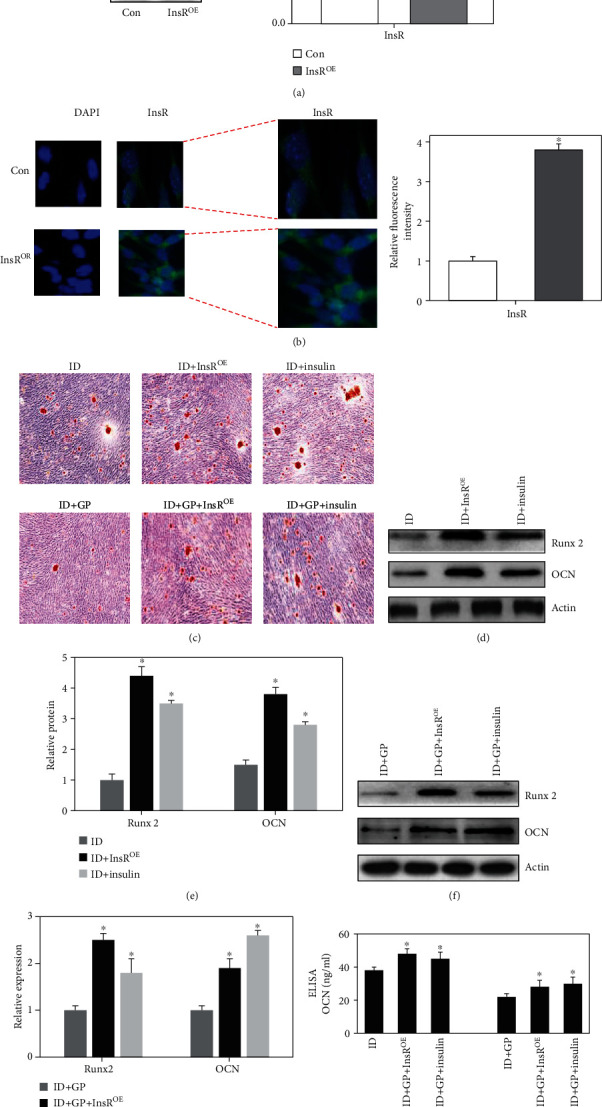
Effect of overexpression insulin receptor (InsR^OE^) on Runx2 and OCN expression. (a) Establishment of insulin receptor overexpression model. (b) The establishment of insulin receptor overexpression model was validated through immunocytochemistry staining. (c) Mineralization was analyzed through alizarin red staining. (d) The protein expression of Runx2 and OCN was measured through western blotting after treatment with ID, ID+InsR^OE^, or ID+Insulin. (e) The protein expression of Runx2 and OCN was analyzed. (f) The protein expression of Runx2 and OCN was measured through western blotting after treatment with ID+GP, ID+GP + InsR^OE^, or ID+GP + Insulin. (h) The protein expression of Runx2 and OCN was analyzed. (g) The level of OCN was measured with ELISA. Compared with the ID or ID+GP group, ^∗^*P* < 0.05. MC3T3-E1 was used in these experiments.

In order to verify the important role of insulin signaling pathway in the process of osteoblast differentiation, we overexpressed the insulin receptor of osteoblast precursor cells (MC3T3-E1). In addition, whether insulin stimulation can stimulate osteoblast differentiation and improve the obstacle of osteoblast differentiation caused by GP was also studied. The successful establishment of insulin receptor overexpression model in the osteoblast precursor cells was verified through western blotting (Figure 3(a)) and immunocytochemistry staining (Figure 3(b)). We found that the mineralized nodules were significantly increased in the group ID+InsR^OE^ and ID+Insulin compared with the group ID (Figure 3(c)). Meanwhile, in the GP-treated model, both InsR^OE^ and insulin significantly promoted the mineralized nodules number (Figure 3(c)). In addition, InsR^OE^ and insulin increased remarkably the protein and mRNA levels of Runx2 and OCN in both ID- and ID+GP-treated models (Figures 3(d)–3(h)). Therefore, overexpression of InsR and insulin can increase the expression levels of osteogenic differentiation indexes including OCN and Runx2 and improve the obstacle of osteoblast differentiation caused by glycolipid toxicity.

### 3.4. The Insulin Increased by CM Overexpressing Insulin Receptor Was Reversed by Osteocalcin Neutralizing Antibody (Anti-OCN)

**Figure 4:**
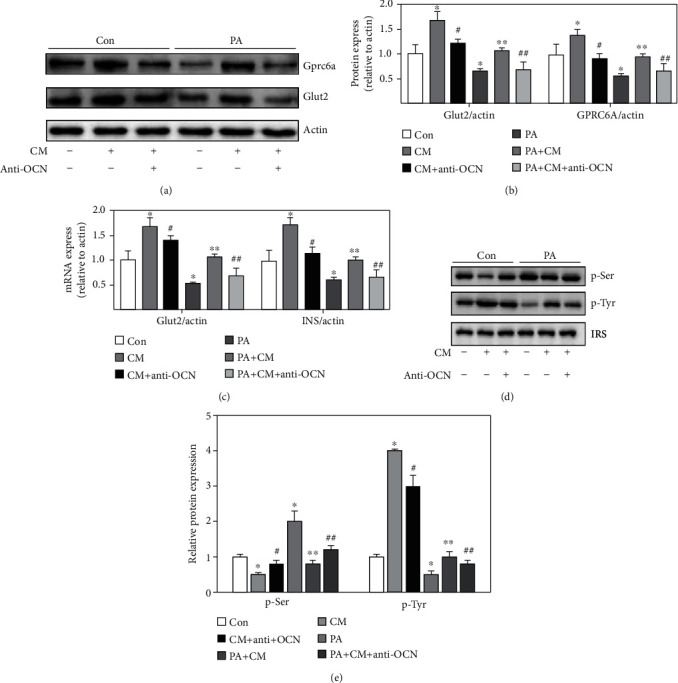
The increase of insulin by CM was reversed by Anti-OCN. (a) The protein expression of Gprc6a and Glut2 was measured through western blotting after treatment with CM or Anti-OCN. (b) The protein expression of Gprc6a and Glut2 was analyzed. (c) The mRNA expression of Gprc6a and Glut2 was measured through qRT-PCR after treatment with CM or Anti-OCN. (d) The serine and threonine phosphorylation of insulin receptor substrate 1 (IRS-1) were measured through western blotting after treatment with CM or Anti-OCN. (e) The serine and threonine phosphorylations of insulin receptor substrate 1 (IRS-1) were analyzed. Compared with the control group, ^∗^*P* < 0.05. Compared with the PA group, ^∗∗^*P* < 0.05. Compared with the CM group, ^#^*P* < 0.05. Compared with the PA + CM group, ^##^*P* < 0.05. Beta-TC6 was used in these experiments.

The influences of CM and Anti-OCN on the expression of GSIS glucose transporters, Gprc6a, and Glut2, in the membrane of islet *β*-cell were investigated. We found that PA remarkably reduced the levels of Gprc6a, Glut2, and insulin compared with the group control (Figures 4(a)–4(c))In addition, the levels of Gprc6a, Glut2, and insulin were remarkably increased by CM, but the increased effects were suppressed by Anti-OCN (Figures 4(a)–4(c)). Therefore, the increase of Gprc6a and Glut2 by CM should be achieved by the OCN secreted by MC3TC-E1 cells overexpressing InsR^OE^. After treatment with anti-OCN, the promotion of Gprc6a, Glut2, and insulin by CM was suppressed. OCN might affect GSIS and insulin through regulating insulin receptor. In addition, the activation of insulin signaling was investigated through measuring the serine and threonine phosphorylation of insulin receptor substrate 1 (IRS-1). We found that CM remarkably promoted threonine phosphorylation but decreased serine phosphorylation compared with the group control (Figures 4(d) and 4(e)). However, the influence of CM on the activation of insulin signaling was reversed by anti-OCN (Figures 4(d) and 4(e)). Meanwhile, the decreased threonine phosphorylation and increased serine phosphorylation induced by PA were reversed by CM (Figures 4(d) and 4(e)).

### 3.5. Undercarboxylated Osteocalcin (ucOC) Suppressed the Lipotoxic Islet *β*-Cell Damage Caused by PA

**Figure 5:**
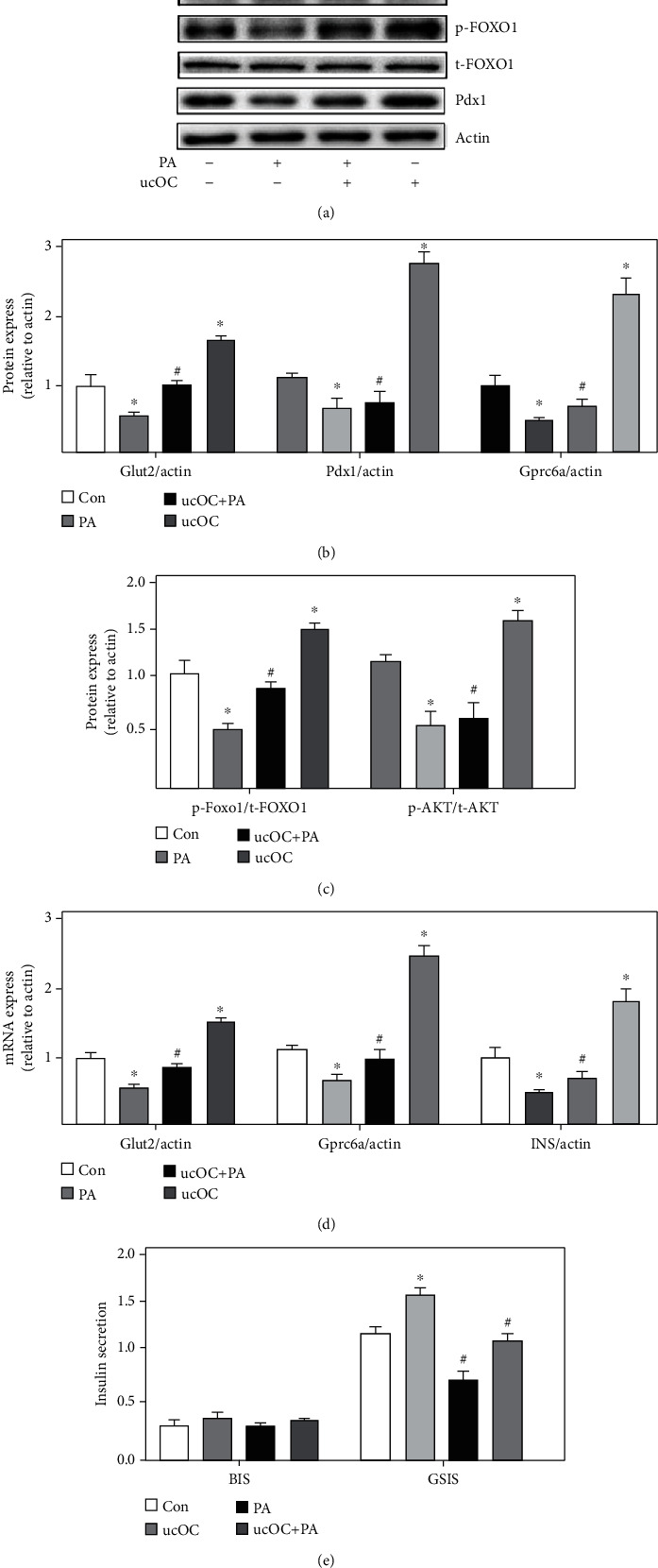
ucOC suppressed the lipotoxic islet *β*-cell damage caused by PA. (a) The protein expression of Gprc6a, Glut2, AKT, FOXO1, and Pdx1 was measured through western blotting after treatment with PA or ucOC. (b) The protein expression of Gprc6a and Glut2 was analyzed. (c) The protein expression of AKT and FOXO1 was analyzed. (d) The mRNA expression of Gprc6a and Glut2 was measured through qRT-PCR. (e) The insulin secretion was analyzed. Compared with the control group, ^∗^*P* < 0.05. Compared with the PA group, ^#^*P* < 0.05. Beta-TC6 was used in these experiments.

The decreased levels of Glut2, Gprc6a, p-AKT/t-AKT, p-FOXO1/t-FOXO1, and Pdx1 caused by PA were significantly increased by ucOC (Figures 5(a)–5(c)). In addition, the results were identified through qRT-PCR (Figure 5(d)). Meanwhile, the decreased insulin secretion induced by PA was also promoted by ucOC (Figures 5(d) and 5(e)). Therefore, ucOC could activate AKT and FOXXO1 and improve insulin secretion disorder caused by lipotoxicity.

### 3.6. Knockdown of Gprc6a or Suppression of PI3K/AKT Signal Pathway Could Reverse the Upregulation of GPRC6A/PI3K/AKT/FoxO1/Pdx1 Caused by ucOC

**Figure 6:**
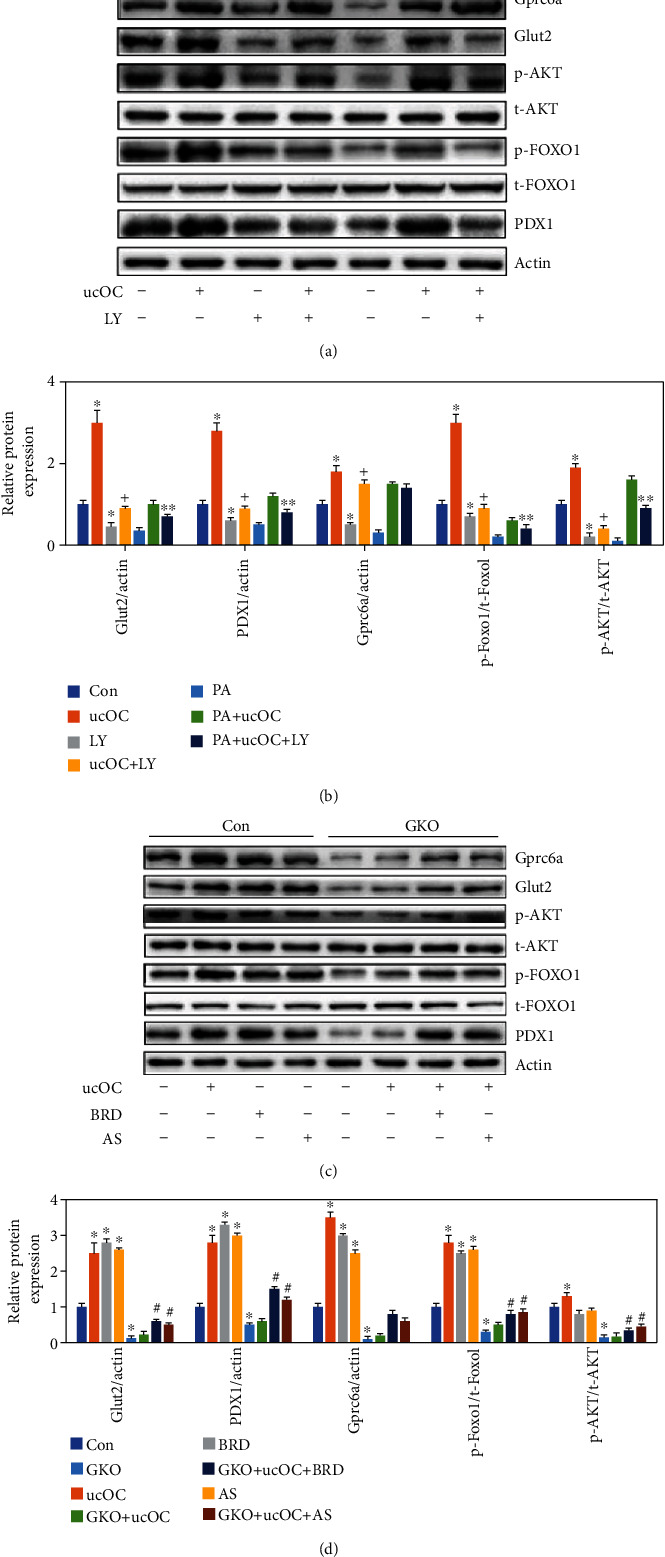
Knockdown of Gprc6a or suppression of PI3K/AKT signal pathway could reverse the upregulation of GPRC6A/PI3K/AKT/FoxO1/Pdx1 caused by ucOC. (a) The protein expression of Gprc6a, Glut2, AKT, FOXO1, and Pdx1 was measured through western blotting after treatment with PA, ucOC, and LY (the inhibitor of PI3K/AKT signal pathway). (b) The protein expression of Gprc6a, Glut2, AKT, FOXO1, and Pdx1 was analyzed. (c) The protein expression of Gprc6a, Glut2, AKT, FOXO1, and Pdx1 was measured through western blotting after treatment with ucOC, BRD (The inducer of Pdx1), and AS (The inhibitor of Foxi1). (d) The protein expression of Gprc6a, Glut2, AKT, FOXO1, and Pdx1 was analyzed. Compared with the control group, ^∗^*P* < 0.05. Compared with the GKO + ucOC group, ^#^*P* < 0.05. Compared with the ucOC group, ^+^*P* < 0.05. Compared with the PA + ucOC group, ^∗∗^*P* < 0.05. Beta-TC6 was used in these experiments.

LY294002 (LY) was used as the inhibitor of PI3K/AKT signal pathway in this study. We found that the levels of Glut2, Gprc6a, p-AKT/t-AKT, p-FOXO1/t-FOXO1, and Pdx1 were significantly inhibited after treatment with LY (Figures 6(a) and 6(b)). In addition, the Gprc6a gene was knocked down in the cells through transfection. After knockdown of Gprc6a, the increase of Glut2 induced by ucOC was blocked (Figures 6(c) and 6(d)), and the levels of Glut2, Gprc6a, p-AKT/t-AKT, p-FOXO1/t-FOXO1, and Pdx1 were significantly suppressed. After supplement with AS1842856 (AS, the inhibitor of Foxi1) or BRD7552 (BRD, the inducer of Pdx1), these inhibition effects were improved.

### 3.7. ucOC Suppressed the Apoptosis of Islet *β*-Cell and Regulated FOXO1 Nuclear Transfer

**Figure 7:**
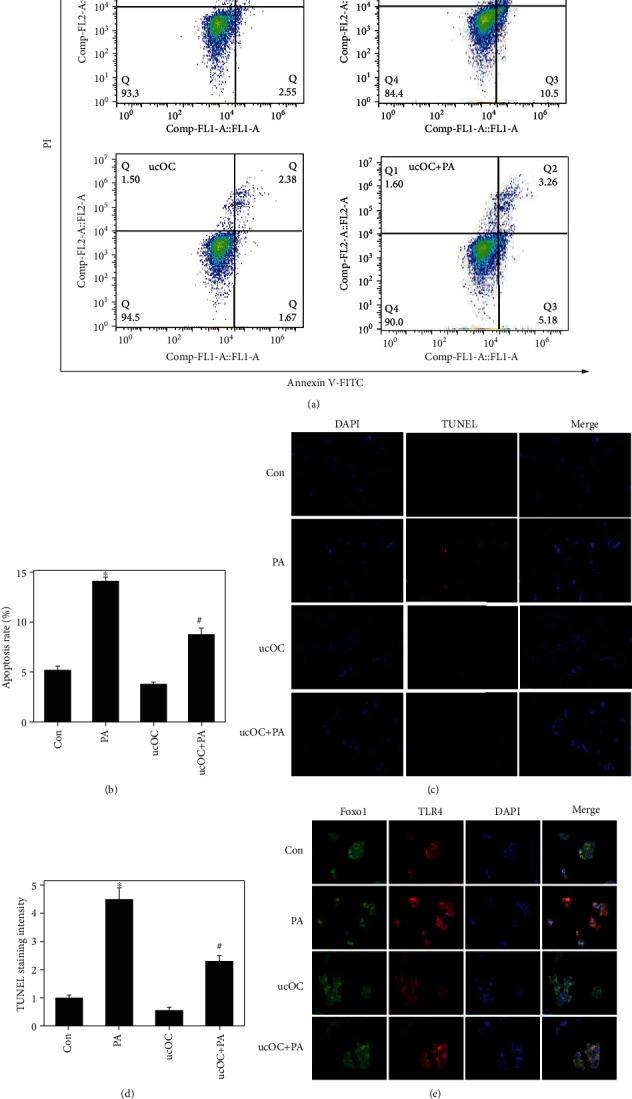
ucOC suppressed the apoptosis of islet *β*-cell and regulated FOXO1 nuclear transfer. (a) The cell apoptosis was measured after treatment with PA or ucOC. (b) The cell apoptosis was analyzed. (c) The cell apoptosis was measured with TUNEL staining. (d) The cell apoptosis was analyzed. (e) The FOXO1 nuclear transfer and TLR4 expression were measured after treatment with PA or ucOC. Beta-TC6 was used in these experiments.

To explore the mechanism of how OCN suppresses glycolipid toxicity and improves insulin, cell apoptosis and FOXO1 nuclear transfer were analyzed. We found that apoptosis rate was significantly reduced from 14.09% in the group PA to 8.44% in the group ucOC+PA (Figures 7(a) and 7(b)). The cell apoptosis was also validated with TUNEL staining. The remarkable increase of cell apoptosis induced by PA was decreased by ucOC (Figures 7(c) and 7(d). Meanwhile, ucOC remarkably promoted the transfer of FOXO1 from intranuclear to extranuclear compared with the group PA and inhibited the expression of TLR4 (Figure 7(e)).

## 4. Discussion

Diabetes is a very common disease and it significantly threats people's health worldwide [1, 2, 31]. The metabolic disorder can be induced by high blood glucose levels or the insulin reduction in the diabetes patients [32]. The differentiation of mature *β*-cell was also an important factor in functional cell reduction and insulin secretion deficiency in patients with diabetes [9, 10]. Our previous studies suggest that chronic exposure to high concentrations of saturated fatty acids, such as palmitic acid (PA) and osteocalcin, could lead to apoptosis of pancreatic *β*-cells [11–13]. Osteocalcin is the bridge between bone metabolism and energy metabolism [14, 15]. Some researchers found that there was no abnormal bone metabolism after selective osteocalcin gene knockout in rats [16]. However, fat thickness, insulin level, glucose tolerance, and other metabolic indicators could be changed, and the real function of this protein has not been well understood [16, 20, 21]. We found that the level of insulin and the expression of GLUT2 were significantly increased in the OCN treatment group compared with the control. Especially, the secretion of insulin was obviously increased. After intervention with exogenous OCN in mice fed with high fat and high glucose, the fat thickness of mice was decreased, but the insulin secretion and sensitivity were increased [22–25].

Previous evidence suggests that *β*-cell dysfunction during the development of T2DM could be regulated by the changes of insulin section [33, 34] and the increase of ceramide, nitric oxide, ROS, and mitochondrial perturbations [35–37]. Several studies have proved that a Fox family transcription factor was involved in the regulation of exocytosis-related gene expression in *β*-cells, especially FOXO1 [38–40]. FOXO1 is a target in the insulin pathway, inhibition of this pathway could reduce exocytotic gene and protein expression, resulting in the dysfunction of cells [39, 41, 42]. In our study, the result showed that the OCN treatment significantly increased the FOXO1 from intranuclear to extranuclear compared with the group PA. Our results are consistent with the view that FOXO1 plays an important role in the gradual decline of *β*-cell function [38]. The PDX1 protein was significantly increased after OCN treatment. The PDX1 protein was an important protein in dedifferentiated *β*-cell [43, 44]. The previous result also showed that the PDX1 expression was increased in islets of T2DM [44]. Based on the above discussion, our results suggest that OCN was an important protein regulating the GLUT2 and PDX1 protein expression in the *β*-TC6 cell.

In summary, our data demonstrated that OCN improved lipotoxic inflammation and apoptosis in *β*-TC6 cells by upregulating the GLUT2 expression and activating FOXO1 pathway. Our results suggested that OCN could activate the FOXO1 signaling pathway to regulate GLUT2 expression and improve the insulin secretion disorder caused by lipotoxicity.

## Data Availability

Data supporting this study has been presented in the manuscript, the data required by editor, reviewer, and reader could be provided by the corresponding author.

## Abbreviations

OCN: Osteocalcin
T2DM: Type 2 diabetes mellitus
InsR^OE^: Overexpression of insulin receptor
ucOC: Undercarboxylated osteocalcin
PA: Palmitic acid
CM: Osteoblast culture medium overexpressing insulin receptor
ID: Induction differentiation
GP: Glucose and palmitic acid
Anti-OCN: Osteocalcin neutralizing antibody
AS: AS1842856
BRD: BRD7552
LY: LY294002.
